# IL-1β, IL-8, and Matrix Metalloproteinases-1, -2, and -10 Are Enriched upon Monocyte–Breast Cancer Cell Cocultivation in a Matrigel-Based Three-Dimensional System

**DOI:** 10.3389/fimmu.2017.00205

**Published:** 2017-03-08

**Authors:** Nancy Adriana Espinoza-Sánchez, Gloria Karina Chimal-Ramírez, Alejandra Mantilla, Ezequiel Moisés Fuentes-Pananá

**Affiliations:** ^1^Unidad de Investigación en Virología y Cáncer, Hospital Infantil de México Federico Gómez, Ciudad de México, México; ^2^Programa de Doctorado en Ciencias Biomédicas, Facultad de Medicina, Universidad Nacional Autónoma de México, Ciudad de México, México; ^3^Departamento de Patología, Hospital de Oncología, Centro Médico Nacional Siglo XXI, Instituto Mexicano del Seguro Social, Ciudad de México, México

**Keywords:** breast cancer, monocytes, matrigel-based 3D culture, inflammation, IL-1β, IL-8, MMPs

## Abstract

Breast cancer remains the first cancer-related cause of death in women worldwide, particularly in developing countries in which most cases are diagnosed in late stages. Although most cancer studies are based in the genetic or epigenetic changes of the tumor cells, immune cells within the tumor stroma often cooperate with cancer progression. Particularly, monocytes are attracted to the tumor primary site in which they are differentiated into tumor-associated macrophages that facilitate tumor cell invasion and metastasis. In this study, we used three-dimensional cultures to form acini-like structures to analyze the inflammatory secretion profile of tumor cells individually or in co-culture with monocytes. Breast cancer cell lines and primary isolates from eight Mexican patients with breast cancer were used. We found high levels of RANTES/CCL5, MCP-1/CCL2, and G-CSF in the breast cancer individual cultures, supporting an important recruitment capacity of monocytes, but also of neutrophils. The co-cultures of the tumor cells and monocytes were significantly enriched with the potent pro-inflammatory cytokines interleukin (IL)-1β and IL-8, known to support malignant progression. We also found that the interaction of tumor cells with monocytes promoted high levels of matrix metalloproteinases (MMP)-1, MMP-2, and MMP-10. Our study supports that a key event for malignant progression is the recruitment of different immune cell populations, which help to sustain and enhance a chronic inflammatory microenvironment that highly favors tumor malignancy.

## Introduction

Breast cancer (BrC) is the most common cancer and the main cause of cancer-related death in women worldwide. BrC greatly affects the quality of life of working-age women, and the likelihood of patients to present aggressive cancer features, such as metastasis, resistance to treatment, and disease relapse, remains high, particularly in developing countries in which BrC is diagnosed at advanced stages (1). Great efforts have been placed to grade BrC into clinical stages to try to improve the effectiveness of therapy schemes. However, tumor classification and directed therapies are based mainly on the genetic or epigenetic changes of the tumor cell itself (2). Today, we understand that tumors are formed by a variety of cells of different origins, the tumor stroma or tumor microenvironment, in which the tumor cell coexists with non-tumor cells, for instance fibroblasts, endothelial cells, and immune cells (3, 4). One of the main cell populations found in the stroma of many types of tumors are the macrophages, termed tumor-associated macrophages or TAMs (5, 6).

Increasing evidence supports that chronic inflammation deeply impacts cancer initiation and progression (7). Circulating monocytes are persistently chemoattracted to inflammatory sites in which they differentiate into macrophages that help to maintain the inflammatory process, thus creating a positive loop. Macrophages were initially thought to exclusively perform immune defense functions, such as removal of invading pathogens and cancer cells, because of their potent phagocytic, oxygen-dependent intracellular killing and antigen-presenting activities. However, macrophages also participate in clearing of aging cells and tissue remodeling in non-pathological conditions and healing or tissue repair after pathological conditions. To do this, macrophages are highly plastic cells of which there are many subtypes expressing different sets of bioactive molecules that contribute to tissue growth through mechanisms of cell proliferation, angiogenesis, and immunosuppression (8–10). Since the pioneering studies by Lin et al. using murine BrC models, it was clear that preventing the arrival of monocytes into the tumor site resulted in small tumors and decreased metastases (11). Today, there is evidence supporting that TAMs have crucial roles in angiogenesis, invasion, and metastasis, and a dense infiltration of these cells into human primary tumors is significantly associated with poor prognosis (12, 13). Both inflammatory and anti-inflammatory macrophages can potentially cooperate with tumor evolution.

To study the communication that tumor cells establish with monocytes, we have previously used aggressive (MDA-MB-231) and non-aggressive (MCF-7) BrC cell lines co-cultured with the monocytic cell line U937 in a Matrigel-based three-dimensional (3D) system, finding that U937 cells significantly upregulate expression of matrix metalloproteinases (MMPs) and inflammatory mediators in response to soluble factors from the most aggressive BrC cells (14). To follow up those findings, we decided to test primary tumor cells obtained from BrC patients and analyze their secretion profiles, both individually and in co-culture with primary monocytes (PM) and monocytic cell lines. We think that working with primary isolates we would obtain information that more closely reflected the biology of the tumors. We found that primary BrC cells are secreting high levels of the chemokines RANTES/CCL5, MCP-1/CCL2, and G-CSF, which revealed their potential capability to recruit and activate monocytes. When BrC cells were co-cultured with monocytes, we observed a significant increased secretion of interleukin (IL)-1β and IL-8, two interleukins associated with malignant progression of several types of cancer. Interestingly, IL-1β and IL-8 also distinguished co-cultures of aggressive from non-aggressive BrC cell lines. Finally, we also observed increased levels of MMP-1, MMP-2, and MMP-10 in co-cultures. This study supports the idea that the potential of aggressiveness of BrC cells is given by their ability to recruit monocytes and to instruct them to secrete high levels of potent pro-inflammatory cytokines IL-1β and IL-8, and metalloproteinases MMP-1, MMP-2, and MMP-10. These mechanisms may favor tumor progression contributing with recruitment of other immune cells and facilitating cell invasion and metastasis.

## Materials and Methods

### Patient Recruitment and Primary Cell Isolation

Patient samples were obtained from the tissue bank of the Unidad de Investigación en Virología y Cáncer, Hospital Infantil de México Federico Gómez, for which patients signed an informed consent, and the protocol was approved by the Scientific, Ethics, and Biosafety Institutional Review Boards. Tumor samples were taken from patients diagnosed with ductal carcinoma and with no previous neoadjuvant therapy before tissue resection.

The tumor tissue was rinsed with PBS and mechanically disaggregated with a scalpel in 1–2 mm fragments, which were subsequently digested for 2 h at room temperature (RT) with a mixture of 1 mg/mL collagenase type I and 100 U/mL hyaluronidase (both from Sigma; Saint Louis, MO, USA) in DMEM/F12 containing 100 U/mL penicillin and 100 μg/mL streptomycin, in constant stirring. The resulting suspension was filtered through a wide pore membrane and subsequently in a 100 μm pore membrane. The cells were pelleted and washed twice with sterile PBS and finally placed in their culture media. Cell morphology of primary isolates was analyzed and compared to the morphology of the commercial BrC cell lines in 2D (monolayer) and 3D culture conditions. Images were taken with an Olympus BX-41 microscope (Olympus Corporation, Tokyo, Japan). Primary cultures (PC) were given consecutive numbers; of 16 tumor tissues plated, eight PC were established for at least seven passages. These cultures were named UIVC-IDC-1, -4, -5, -6, -9, -10, -11, and UIVC-NIDC-1.

### Cell Culture

All commercial cell lines were obtained from the American Type Culture Collection (ATCC, Manassas, VA, USA) and culture media and supplements from Gibco Invitrogen Cell Culture (Carlsbad, CA, USA) unless specified differently. The human monocytic cell lines THP-1 (No. TIB-202), U937 (No. CRL-1593.2), and the BrC line T47D (HTB-133) were cultured in RPMI 1640 medium supplemented with 10% fetal bovine serum (FBS), 100 U/mL penicillin, and 100 U/mL streptomycin, at 37°C in 5% CO_2_. BrC cells MCF-7 (No. HTB-22) and MDA-MB-231 (No. HTB-26) were cultured in DMEM/F12 medium supplemented with 10% FBS, 100 U/mL penicillin, and 100 μg/mL streptomycin, at 37°C in 5% CO_2_. BrC cells HS578T (No. HTB-126) were cultured in high glucose (4.5 g/L) DMEM medium supplemented with 10% FBS, 100 U/mL penicillin, and 100 μg/mL streptomycin, at 37°C in 5% CO_2_. MCF-10A cells (No. CRL-10317) were cultured in DMEM/F-12 medium supplemented with 20 ng/mL of epidermal growth factor (EGF; PeproTech, Rocky Hill, NJ, USA), 10 μg/mL insulin, 0.5 μg/mL hydrocortisone, 100 ng/mL cholera toxin (all from Sigma Chemical Co., St. Louis, MO, USA), 5% horse serum, 100 U/mL penicillin, and 100 μg/mL streptomycin. Primary tumor cell isolates were cultured in DMEM/F12 supplemented with 5% horse serum, 100 U/mL penicillin and 100 μg/mL streptomycin, 100 ng/mL cholera toxin, 0.5 μg/mL hydrocortisone, 10 μg/mL insulin, and 20 ng/mL of EGF at 37°C in 5% CO_2_ atmosphere.

### Isolation of Peripheral Blood PM

Peripheral blood mononuclear cells (PBMCs) were isolated according to the following protocol: 40 mL of blood of healthy volunteers was extracted, diluted in a 1:3 proportion with sterile endotoxin-free PBS (Gibco Invitrogen Cell Culture), and subjected to density gradient centrifugation with Histopaque^®^-1077 (Sigma-Aldrich Inc., St. Louis, MO, USA) per 30 min at 2,000 rpm. PBMCs were then carefully retrieved from the gradient and washed three times with PBS, each time followed by slower centrifugation (1,500, 1,250, and 1,000 rpm). To obtain the monocyte-enriched fraction, PBMCs were subjected to negative selection with the Monocyte Isolation Kit II Human (Miltenyi Biotec Inc., Auburn, CA, USA) following the manufacturer’s recommendations as we briefly describe next. PBMCs were washed once with diluted 1:20 MACS BSA Stock Solution (Miltenyi Biotec Inc., Auburn, CA, USA), counted, and adjusted to a density of 10^7^ cells per 30 μL of buffered solution; 10 μL of FcR-blocking reagent and 10 μL of monocyte biotin-antibody cocktail were then added for every 10^7^ cells to be labeled; cells were mixed and incubated for 15 min at 4°C. An additional 30 μL of buffered solution was added plus 20 μL of anti-biotin microbeads for every 10^7^ cells to be labeled; cells were mixed and incubated for 20 min at 4°C. Cells were then washed once with buffered solution, centrifuged at 1,500 rpm for 5 min, and resuspended in 1.5 mL of buffered solution for magnetic separation. Suspension of cells was passed through a pre-rinsed LS column (Miltenyi Biotec Inc., Auburn, CA, USA) and 7 mL of buffered solution was added. The monocyte-enriched cells were collected in a conical 15 mL tube, counted, and if not cultured immediately, monocytes were frozen at a density of 2 × 10^6^ in 1 mL of DMEM/F12 medium supplemented with 50% FBS and 10% DMSO at −80°C. PM used for experimentation were never frozen for more than 2 months after isolation. Cultures were maintained in DMEM/F12 medium supplemented with 6% FBS, 100 U/mL penicillin, and 100 μg/mL streptomycin, at 37°C in 5% CO_2_ atmosphere. Each set of experiments was performed utilizing PM of at least two different donors (each donor isolates independently because pooling resulted in monocyte activation). The phenotype of purified monocytes was very homogeneous between different isolates: CD34^neg^ CD11b^pos^ CD14^pos^ CD64^pos^ CD68^neg^ CD16^neg^, and also with both monocytic cell lines, which corresponds to the phenotype of a non-activated immature monocyte (15).

### Immunocytochemistry of Epithelial Markers

A total of 20,000 cells of the primary isolates were plated on 8-well chamber slides (Nunc^®^ Lab-Tek^®^ Chamber Slide System, Sigma), cultured for 48 h in their correspondent media, rinsed with PBS, and fixed with paraformaldehyde at RT for 10 min. Cells were then hydrated with PBS and permeabilized with 0.05% triton X-100 for 10 min at 4°C. Endogenous peroxidase activity was blocked by incubating the slides in peroxidase blocking solution (Dako, Inc., Carpinteria, CA, USA). Non-specific antibody binding was blocked by incubation with 8% albumin in PBS for 20 min. The slides were incubated with the following primary antibodies at an optimized concentration: mouse monoclonal anti-human anti-PanCytokeratin (Clone AE1/AE3; Biocare Medical; Concord, CA, USA), mouse monoclonal anti-human anti-mucin 1 (MUC-1) [epithelial membrane antigen (EMA; Clone Mc-5; Biocare Medical)], and mouse monoclonal anti-human anti-epithelial cell adhesion molecule (EpCAM, clone AUA1; Biocare Medical). Cells were incubated overnight in a moist chamber at 4°C. The EnVision Detection Kit (Dako; Carpinteria, CA, USA) was employed as the detection system. Cells were counterstained with hematoxilin for 5 min followed by 5 min 0.9% ammonium hydroxide at RT, rinsed with distilled water, and left to dry at RT and permanently cover slipped. Slides were analyzed and photographed with an Olympus BX-41 microscope (Olympus Corporation, Tokyo, Japan).

### Primary Culture Growth in Medium of Mesenchymal Cell

4 × 10^5^ cells from primary isolates were seeded in 4 mL of low glucose (1 g/L) DMEM medium (Gibco Invitrogen), with sodium pyruvate 110 mg/L, without HEPES and supplemented with 15% FBS, 1% l-glutamine, and 100 U/mL penicillin and 100 μg/mL streptomycin and incubated at 37°C in 5% CO_2_. Cells were maintained for 5–7 days in the described conditions and their growth evaluated. Results were scored as *none* when no growth was observed, *one cross* when cells attached and formed ≈40% of a confluent layer, *two crosses* 80–90% of confluence, and *three crosses* when cells were passaged and proliferate in a new culture flask with mesenchymal cell medium. Bone marrow mesenchymal cells were used as positive control and MCF-10A cells as negative control.

### 3D Culture and Harvest of Cell Culture Supernatants

For 3D individual cultures, a 40 μL base of Matrigel was added per well of an 8-well chamber slide system (8-well plates, Lab-Tek Chamber Slide System, Nalgene Nunc International, Rochester, NY, USA), incubated for 30 min at 37°C and 800 cells were added in 400 μl of the correspondent culture medium supplemented with 4 ng/mL of EGF and 2% Matrigel. For 3D co-cultures, 4 × 10^5^ monocytes contained in 1 mL of their correspondent medium supplemented with 2% Matrigel and 2% FBS for U937 and THP-1 monocytes, or 2% Matrigel and 6% FBS for PM, were plated per well of a 24-well flat-bottom culture plate. A transwell cell culture insert with pore size of 0.4 μm (Thermo Fisher Scientific™ Nunc^TM^; Waltham, MA, USA) was placed in each well containing 1 mL of a 4 × 10^5^ BrC cells in their correspondent medium supplemented with 2% Matrigel and 2% FBS (for commercial cell lines) or 5% horse serum for PC. Controls of individual cell cultures with the same media were included. After 5 days of culture, the supernatants from bottoms and tops of the 3D co-cultures were recovered, mixed, aliquoted, and kept at −20°C until use.

### Analysis of Cytokine Profiles

The following analytes were determined in the supernatants of the cultures: granulocyte-colony-stimulating factor (G-CSF), granulocyte-macrophage-colony-stimulating factor (GM-CSF), interleukin (IL)-1 beta (IL-1β), IL-2, IL-4, IL-6, IL-8, IL-10, IL-12p70, IL-17, interferon-alpha 2 (INF-α2), monocyte chemoattractant protein-1 (MCP-1) also known as chemokine CCL2, regulated on activation normal T cell expressed and secreted (RANTES) also known as chemokine CCL5, EGF, vascular endothelial growth factor (VEGF), and a panel of the MMP-1, -2, -7, -9, and -10. The determinations were done with the multiplexing assay platform from MILLIPLEX (EMD Millipore Corporation, Billerica, MA, USA) following the manufacturer’s recommended procedure. Briefly, in each well of a 96-well flat-bottom culture plate 25 μL of assay buffer was mixed with 25 μL of supernatants or controls and 25 μL of the detection microbeads cocktail. The mixture was incubated at 4°C overnight with orbital agitation. Wells were then washed twice with washing buffer, 25 μL of the detection antibodies mix was added to each well, and the plate was incubated at RT with orbital agitation for 1 h. After incubation, 25 μL of streptavidin-phycoerythrin was added to each well followed by 30 more minutes of incubation at RT with orbital agitation. The wells were then washed twice with washing buffer, 150 μL of PBS was added to each well to proceed with the analysis in Luminex MAGPIX multiplexing instrument, and the analysis of data was performed in the xPONENT^®^ Software. Three biological replicates were analyzed.

### Migration and Invasion Assays

Migration assays with U937, THP-1, and fresh PM were performed in 24-well plates using polycarbonate membrane transwell inserts with 8-μm pores (Corning Costar, USA) filled with Matrigel. 1.5 × 10^5^ monocytes were resuspended in 200 μL of RPMI without serum and placed in the upper chamber. Then, transwells were placed in a 24-well culture dish containing 800 μL of RPMI without serum but supplemented with either 100 ng/mL of GM-CSF, MCP-1, or RANTES (all from PeproTech, Rocky Hill, NJ, USA) that was used as chemoattractant. Cell migration was allowed to progress for 24 h at 37°C in a humidified 5% CO_2_ environment. The migrating cells were counted at 2, 4, 6, and 24 h using a microscope Motic AE31, and images were acquired with a digital camera (Moticam 5.0 MP). The mean cell count from three random fields (at 100× magnification) was used for the analysis. For the BrC cell lines invasion assays, a total of 6 × 10^5^ cells were resuspended in 200 μL of their respective media without sera and placed in the upper chamber of the Transwell filled with Matrigel (as for the monocytes). The Transwells were placed in a 24-well culture dish containing 800 μL of their respective media with sera or without sera but supplemented with 100 ng/mL of IL-8 as chemoattractant (PeproTech, Rocky Hill, NJ, USA). The experiment proceeded as for monocytes and after 48 h, invading cells were stained with crystal violet and observed under the microscope. The mean cell count from three random fields (at 100× magnification) was used for the analysis of Figure 6C and the integrated optical density (IOD) for the analysis of Figure S1 in Supplementary Material. Three biological replicates were performed.

### Immunofluorescence Assay

3 × 10^4^ cells of each of the cell lines were seeded on coverslips for 24 h in their respective media. Then, cells were fixed with 4% paraformaldehyde for 10 min, and permeabilized with 0.2% Triton X-100 in PBS for 20 min. Cells were blocked with blocking buffer (10% goat serum, 1% BSA, 0.2% triton X-100, and PBS 1X) for 1 h and then stained with the primary antibodies: mouse monoclonal anti-E-Cadherin antibody (Clone: 36/E-cadherin; BD Biosciences, San José, CA, USA), rabbit monoclonal anti-Vimentin antibody-Alexa Fluor-594 (Clone: EPR3776; Abcam, Cambridge, MA, USA), overnight at 4°C. After that, cells were incubated with the secondary antibody goat anti-mouse-IgG-FITC antibody (Sigma-Aldrich Co., St. Louis, MO, USA) for 30 min. Nuclei were stained with DAPI for 25 min. Cells were observed using a fluorescence microscope Olympus BX51, and images were acquired with a digital camera (Camedia C4040, Olympus).

### Statistical Analysis

Statistical comparison of values from the different conditions tested was performed with the GraphPad Prism 5 Software, using Kruskal–Wallis test and Mann–Whitney test to compare all data columns. Statistical significance (*p*) ≤0.05 was indicated with *, ≤0.01 was indicated with **, and ≤0.001 with ***.

## Results

### Aggressive Breast Cancer Cells Establish a Microenvironment Enriched with IL-1β and IL-8

We have previously generated evidence that aggressive BrC cells modulate the expression of monocytes to favor conditions for tumor invasion (14). We define the aggressive potential of BrC cells as follows: to present a cancer stem cell-like phenotype CD44^+^ CD24^−/low^; to present the epithelial-to-mesenchymal transition (EMT), which is characterized by positivity to vimentin (mesenchymal marker) and negativity to E-cadherin (epithelial marker), and to be invasive in transwell assays. According to these characteristics, MCF-7 and T47D cells were classified as non-aggressive and MDA-MB-231 and HS578T cells as aggressive. This characterization correlates with the ability of MDA-MB-231 and HS578T cells, but no of MCF-7 and T47D cells, to form metastasis in immunodeficient mice (16–19). To extend our initial observations, we used three different types of monocytes: commercial U937 and THP1 monocytes, which were obtained from patients with diffuse histiocytic lymphomas and have been widely used as models of macrophage differentiation (20–23), and PM isolated from healthy donors. All the different monocytes presented an immature non-activated phenotype (15).

**Figure 1:**
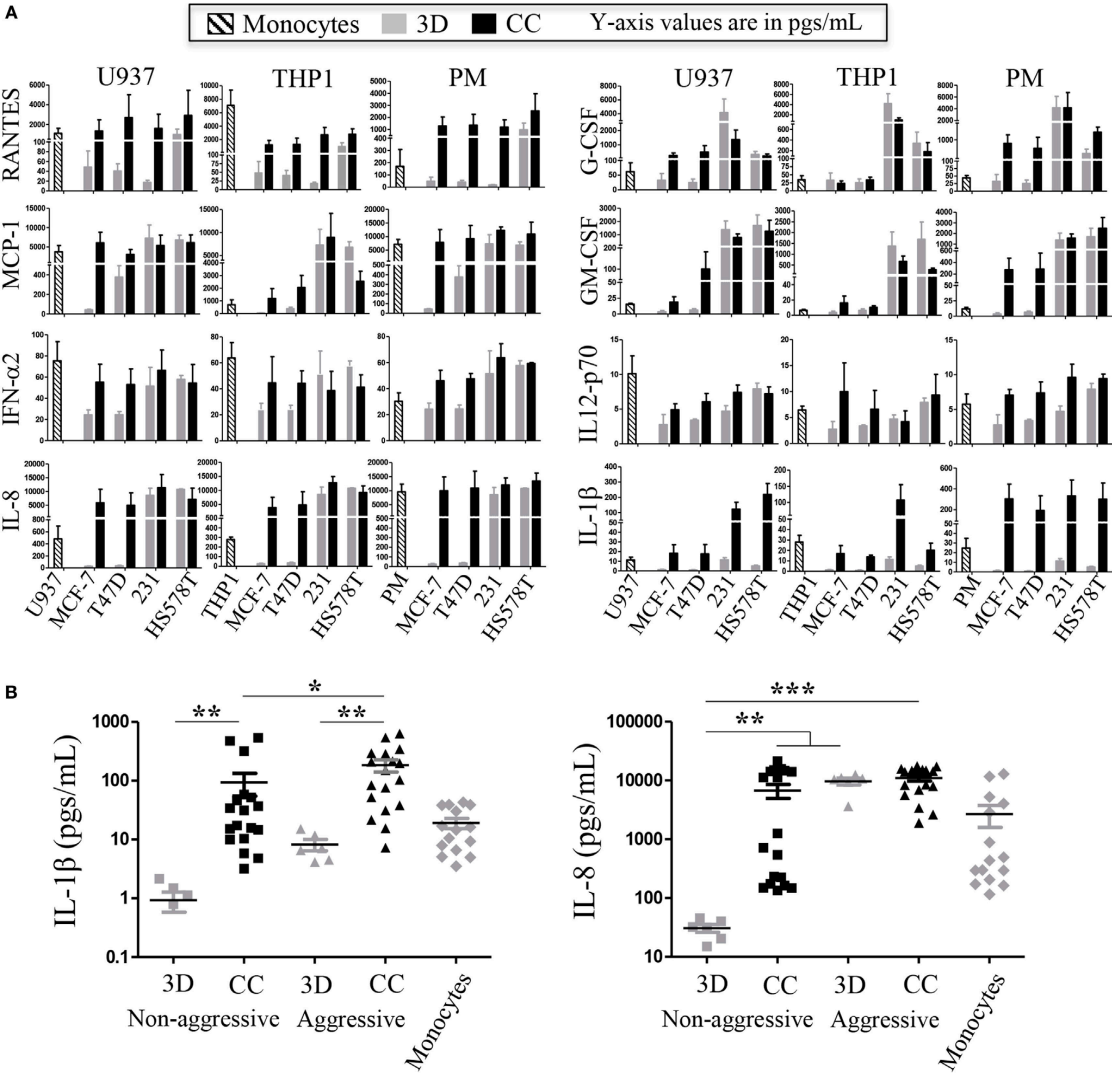
**Levels of inflammatory cytokines present in supernatants of commercial breast cancer (BrC) cell lines in co-culture with monocytes**. Inflammatory cytokines were determined in three-dimensional (3D) individual cultures of the BrC cell lines (indicated as 3D) and in co-culture with the three different sources of monocytes independently [THP-1, U937, and primary monocytes (PM); indicated as cells co-cultured (CC)]. **(A)** The means and SD of three independent co-culture experiments are shown (in picogram/milliliter). **(B)** Summary of the cytokines that gave statistically significant differences. For the statistical analysis, MCF-7 and T47D cells were grouped as *non-aggressive BrC cells* and MDA-MB-231 and HS578T were grouped as *aggressive BrC cells*. THP-1, U937, and PM were grouped as *Monocytes*, and statistics were calculated using all combinations of aggressive vs non-aggressive BrC cells with all the types of monocytes. Left panel [interleukin (IL)-1β]: statistical differences were found between BrC cells cultured individually vs in co-culture with monocytes, and between co-cultures of the non-aggressive BrC cells vs the co-cultures of the aggressive cells. Right panel (IL-8): statistical differences were found between non-aggressive cells cultured individually (3D) and the rest of the groups: non-aggressive CC with monocytes and with aggressive cells either cultured individually or in co-culture with monocytes. Statistical significance *p* ≤ 0.05 was indicated with *, *p* ≤ 0.01 with **, and *p* ≤ 0.001 with ***. The statistical tests comparing monocytes against co-cultures showed significant differences only for HA-BrC (IL-1β *p* ≤ 0.0001 and IL-8 *p* = 0.0014) but not for NA-BrC cells (IL-1β *p* = 0.752 and IL-8 *p* = 0.0942).

The co-cultures with the commercial BrC cell lines and monocytes were done in a Matrigel 3D matrix, after which the content of several cytokines, chemokines, and growth factors (G-CSF, GM-CSF, IL-1β, IL-2, IL-4, IL-6, IL-8, IL-10, IL-12p70, IL-17, INF-α2, MCP-1, RANTES, EGF, and VEGF) were measured in the supernatants. Consistent readings were obtained for the following analytes: RANTES, MCP-1, G-CSF, GM-CSF, IL-8, IL-1β, INFα2, and IL12p70, all related to inflammatory processes, although only the first six have been previously associated with pro-tumor effects. Figure 1A shows three independent assays of the three types of monocytes in co-culture with the commercial BrC cells. It can be observed that IL-1β and IL-8 levels were increased upon co-culture, while the opposite happened with INF-α2 and IL-12p70, which presented the highest levels in some of the monocytes cultured individually. The most relevant result observed was that of IL-1β and IL-8 (Figure 1B). In the case of IL-1β, we found statistical differences between co-cultures and individual cultures. Interestingly, the concentration of IL-1β also allowed distinguishing between co-cultures of the most aggressive BrC cells (average of 183.7 pg/mL) and the non-aggressive cells (94.0 pg/mL). Similarly, IL-8 basal levels (3D) perfectly segregate non-aggressive (average of 30.83 pg/mL) from aggressive tumor cells (average of 9,654 pg/mL). Also, the levels of IL-8 increased significantly when non-aggressive tumor cells were co-cultured with monocytes (average of 6,737 pg/mL), although we did not observe a significant difference between co-cultures of the BrC aggressive cells and co-cultures of the non-aggressive cells.

### Clinical Characteristics of Breast Cancer Patients and Morphology of Cell Isolates in Culture

To extend these observations, the experiments were repeated using primary tumor cells isolated from BrC Mexican patients. The tumor cells were derived from eight female patients with an average age of 56.8 years (range 42–75). All tumors were classified as ductal, with seven out of eight already showing tissue infiltration and three patients already presenting lymph node involvement. Seven tumors were classified as histological grade II and one as grade III, and seven were clinical stages IIA or IIB and one stage I (Table 1).

**Table 1:** **Clinical characteristics of breast cancer patients**.

Primary culture	Age	Histological subtype	Histological grade	TNM staging	Clinical stage
UIVC-IDC-1	52	IDC	2	T1N0M0	I
UIVC-IDC-4	63	IDC	2	T1N1M0	IIA
UIVC-IDC-5	75	IDC	2	T2N0M0	IIA
UIVC-IDC-6	55	IDC	2	T1N0M0	IIA
UIVC-NIDC-1	56	DCIS	2	T1N0M0	IIA
UIVC-IDC-9	42	IDC	2	T2N1M0	IIB
UIVC-IDC-10	64	IDC	3	T3N0M0	IIB
UIVC-IDC-11	48	IDC	2	T2N1M0	IIB

*IDC, invasive ductal carcinoma; DCIS, ductal carcinoma in situ*.

**Figure 2:**
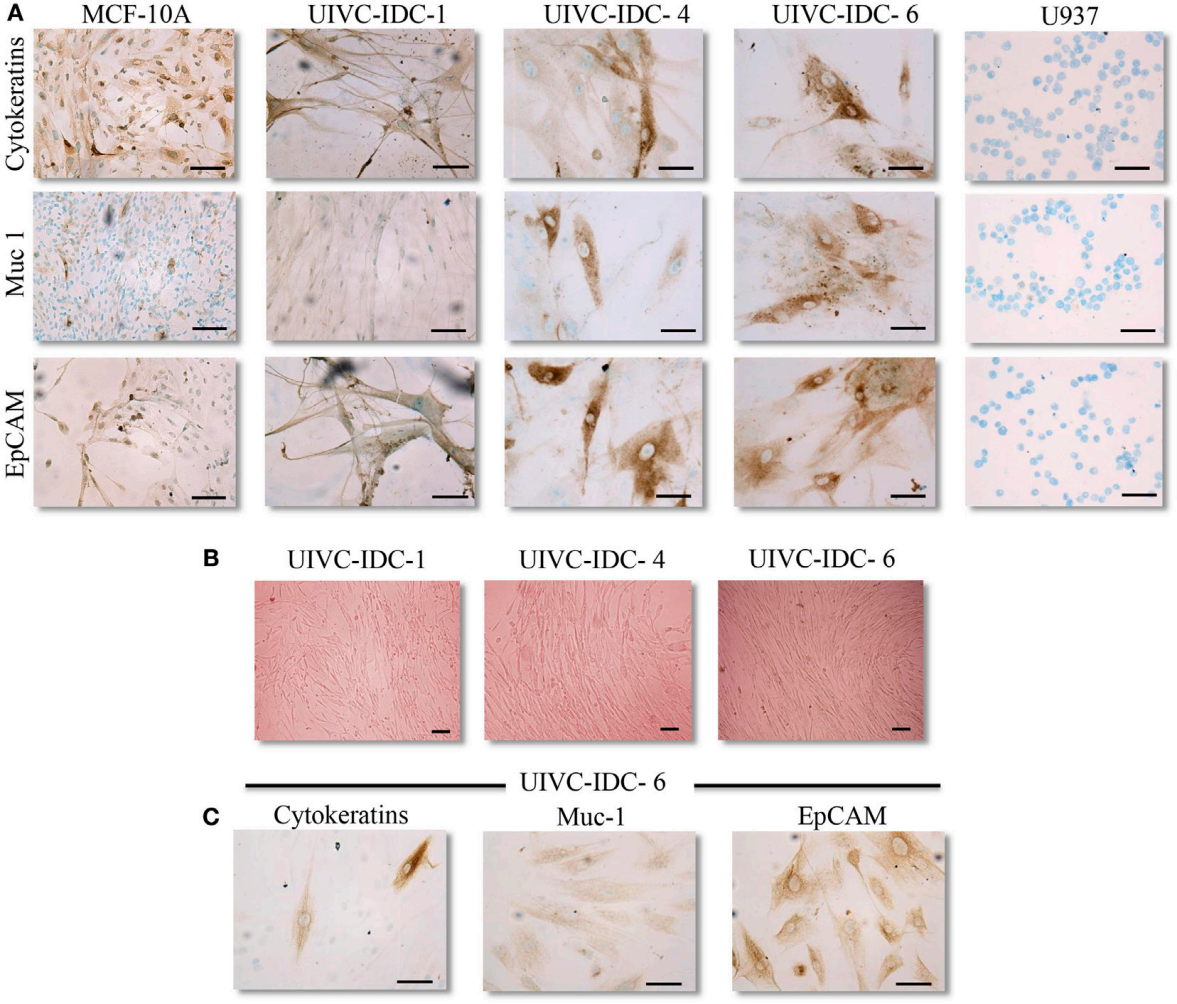
**Epithelial markers in breast cancer primary cell cultures**. **(A)** Representative images of epithelial markers (PanCytokeratin, Muc-1/epithelial membrane antigen, and EpCAM) in three primary cultures (PC) determined by immunocytochemistry. Non-transformed MCF-10A breast epithelial cells were used as positive and U937 monocytes as negative staining controls. **(B)** Representative images of PC UIVC-IDC-1, -4, and -6 proliferating in mesenchymal medium (magnification 100×). **(C)** Epithelial markers determined by immunocytochemistry in UIVC-IDC-6 after one passage in mesenchymal medium. Scale bars correspond to 100 μm, magnification 200×.

For the duration of this study, PC were maintained for up to seven passages in mammary epithelial cell growth medium. We determined their identity as epithelial cells after positive staining to three well known epithelial markers: a panel of cytokeratins, mucin 1, and EpCAM (Figure 2A; Table 2). The primary tumor cells expanded in epithelial cell growth medium were also plated in medium specialized to grow mesenchymal cells, we thought that this was important to exclude the possibility of culture contamination with other resident cells of the tumor stroma that could have pro-tumoral activities. We found that cultures UIVC-IDC-1, -4, -6 and UIVC-NIDC-1 proliferated to different extents in mesenchymal medium (Figure 2B; Table 2). However, cells recovered after expansion in this medium still were positive for epithelial markers (Figure 2C). We concluded that cells expanding in this medium were not mesenchymal cells contaminating the primary isolates, but rather that these tumor epithelial cells acquired mesenchymal characteristics that allowed them to adapt to mesenchymal culture conditions. MCF-10A non-transformed cells were unable to expand in mesenchymal medium (not shown).

**Table 2:** **Characterization of breast cancer primary cultures**.

Primary culture	Epithelial markers	Proliferation in epithelial medium	Proliferation in mesenchymal medium	Growth (2D)	Growth three-dimensional	EMT	Invasion
UIVC-IDC-1	Pos	Pos	++	Aggressive	Aggressive	ND	ND
UIVC-IDC-4	Pos	Pos	++	Aggressive	Aggressive	ND	ND
UIVC-IDC-5	Pos	Pos	None	Non-aggressive	Aggressive	ND	ND
UIVC-IDC-6	Pos	Pos	+++	Aggressive	Aggressive	Pos	Pos
UIVC-NIDC-1	Pos	Pos	+	Non-aggressive	Aggressive	Pos	Pos
UIVC-IDC-9	Pos	Pos	None	Aggressive	Aggressive	Pos	Pos
UIVC-IDC-10	Pos	Pos	None	Non-aggressive	Aggressive	Pos	Pos
UIVC-IDC-11	Pos	Pos	None	Aggressive	Aggressive	ND	ND

*EMT, epithelial to mesenchymal transition; ND, non-determinated; Pos, positive*.

**Figure 3:**
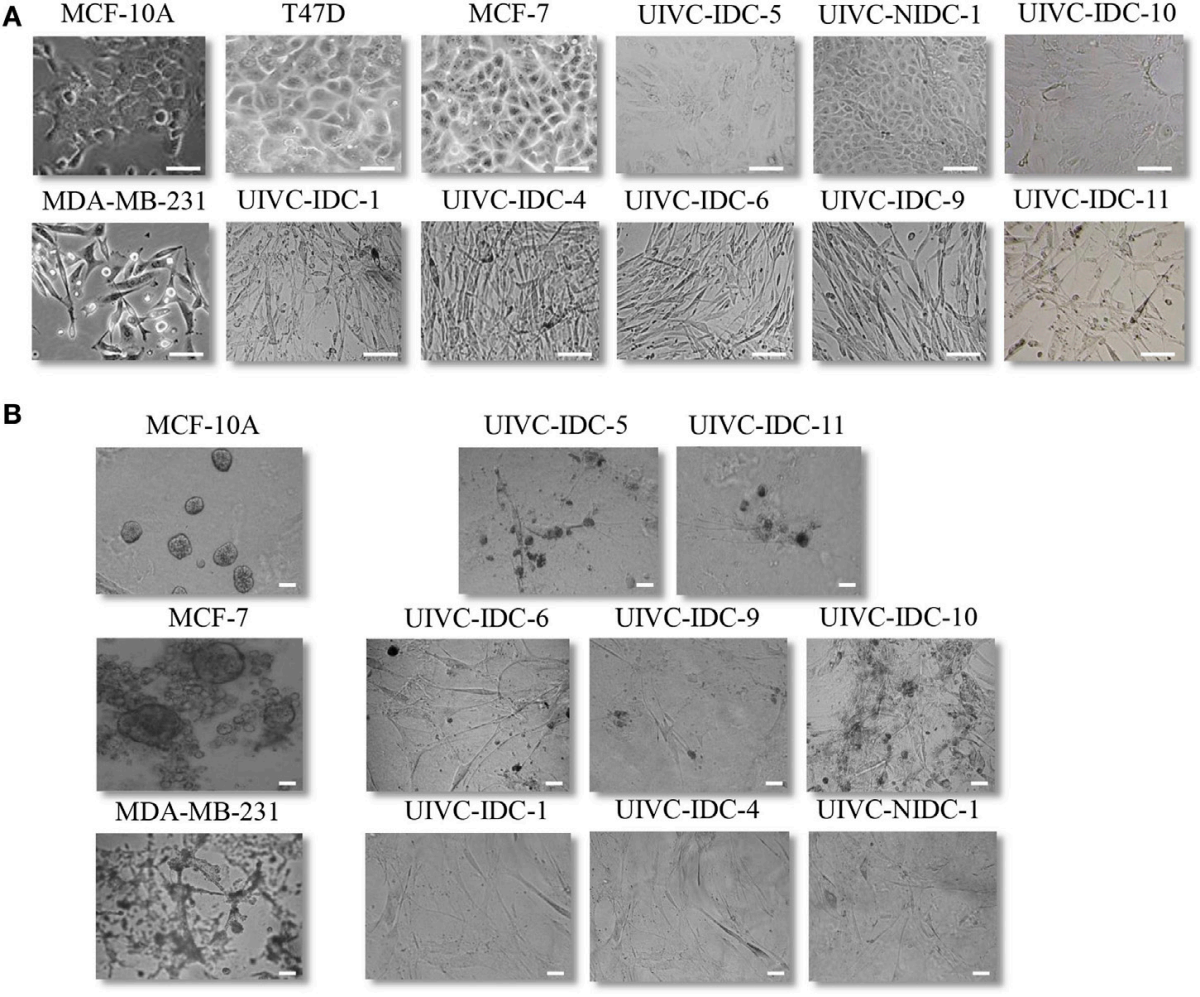
**2D and three-dimensional (3D) growth of breast cancer (BrC) primary cultures (PC)**. **(A)** MCF-7 and T47D cobblestone-like growth was used as representative of cells with non-aggressive features, while MDA-MB-231 and HS578T spindle-like growth was used as representative of aggressive BrC cells. MCF-10A cells are included as the type of growth observed with non-transformed cells. Representative images of PC UIVC-IDC-5, UIVC-NIDC-1, and UIVC-IDC-10 in early passages, which displayed a very similar morphology to non-aggressive BrC cells, while the rest of the primary isolates showed a 2D morphology closer to the one found in aggressive cells. Scale bars correspond to 100 μm, magnification 200×. **(B)** Comparison of the 3D growth of primary isolates with commercial non-transformed, aggressive, or non-aggressive cell lines. Scale bars correspond to 50 μm, magnification 200×.

We assessed the morphology of cells in monolayer (2D) according to the capacity to form cobblestone or spindle-like shapes (Figure 3A; Table 2). Only culture UIVC-IDC-5, -10 and UIVC-NIDC-1 formed an organized monolayer with polygonal-shaped cells and well-delimited cell-to-cell interactions. Cells from the rest of the cultures had an elongated shape, formed less organized monolayers and in some regions cells stacked randomly on top of each other, a characteristic of cells that have lost contact inhibition. We also compared the 3D colonies of the primary isolates with the ones formed by MCF-10A, and the non-aggressive and aggressive cell lines. MCF-10A non-transformed cells form well-organized spherical structures in which cells are polarized with the basal side facing the extracellular matrix (ECM) and the basolateral side facing the hollow lumen of the sphere. Only cultures UIVC-IDC-5 and -11 formed medium size structures resembling spheres, cultures UIVC-IDC-6, -9, and -10 formed smaller structures and cultures UIVC-IDC-1, UIVC-IDC-4, and UIVC-NIDC-1 did not organized in any kind of recognizable sphere-like shape (Figure 3B; Table 2). Based on the 2D and 3D morphology, we conclude that the primary isolates more closely resemble the highly aggressive BrC cells, which is in contrast with most of the patients’ clinical profiles.

### Co-cultures of Primary Breast Cancer Isolates and Monocytes Are Also Enriched with IL-1β and IL-8

**Figure 4:**
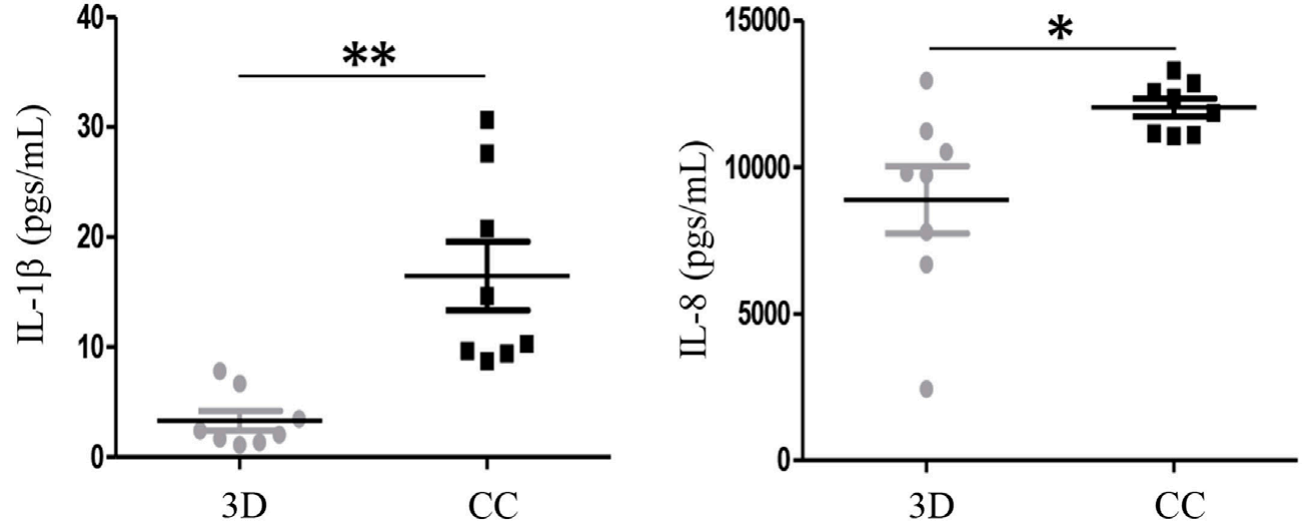
**Levels of interleukin (IL)-1β and IL-8 increased when primary breast cancer (BrC) cells were co-cultured with monocytes**. Levels of IL-1β (left panel) and IL-8 (right panel) significantly increased when primary cells derived from the eight BrC patients were co-cultured with the primary monocytes from healthy donors. Three independent experiments were carried out and each point represents the mean of all determinations for each BrC primary isolate. Statistical significance *p* ≤ 0.05 is indicated with * and *p* ≤ 0.01 with **.

We co-cultured the primary BrC isolates with the PM. The results showed that MCP-1, G-CSF, RANTES, GM-CSF, INF-α2, and IL12-p70 analytes gave no differences between the 3D individual cultures and the 3D co-cultures (data not shown). However, IL-1β and IL-8, which in commercial lines allowed to distinguish between aggressive and non-aggressive BrC cells, showed again a significant increased concentration in co-culture (Figure 4). IL-1β increased from 3.3 pg/mL (individual 3D culture) to 16.5 pg/mL (co-culture), while IL-8 increased from 8,886.3 pg/mL (3D) to 12,027.4 pg/mL (CC).

### MMP-1, MMP-2, and MMP-10 Are Induced in Breast Cancer Primary Cells/Monocytes Co-Cultures

**Figure 5:**
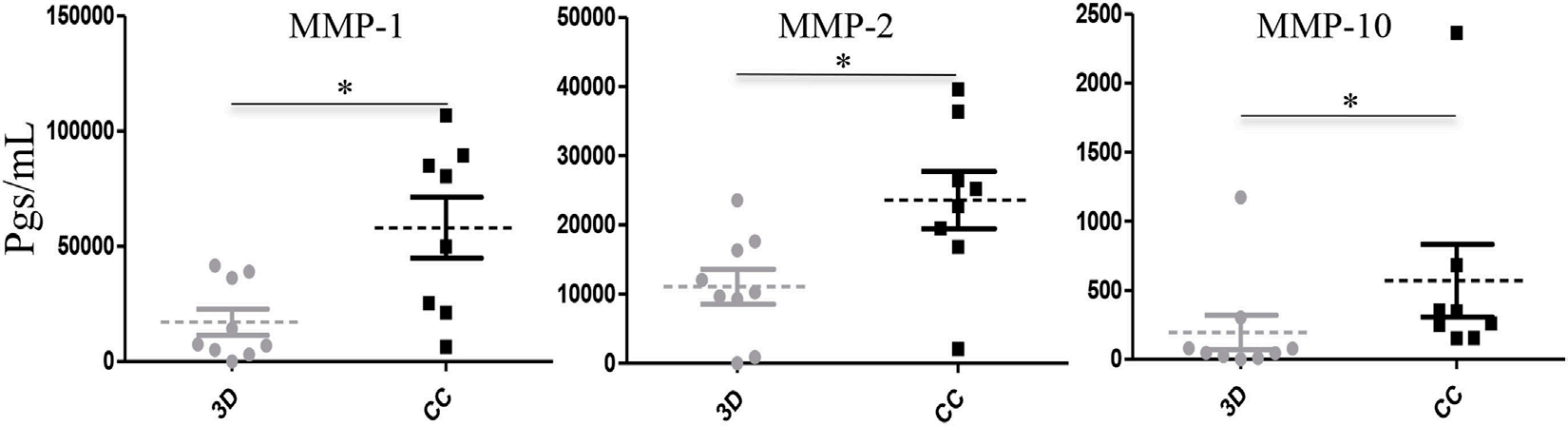
**Matrix metalloproteinases (MMP)-1, MMP-2, and MMP-10 are induced in breast cancer (BrC) primary cell/monocytes co-cultures**. Concentrations of MMP-1, MMP-2, and MMP-10 measured in supernatants significantly increased when primary cells derived from the eight BrC patients were co-cultured with the primary monocytes from healthy donors. Three independent experiments were carried out, and each point represents the mean of all determinations for each BrC primary isolate. Statistical significance (*p* ≤ 0.05) is indicated with *.

Our initial findings also support that aggressive MDA-MB-231 cells promote secretion of MMPs in co-culture with U937 monocytes (14). To extend those results, we analyzed the levels of MMP-1, MMP-2, MMP-7, MMP-9, and MMP-10 present in the primary isolates individually cultured or co-cultured with PM. We did not find significant differences in the secretion levels of MMP-7 and MMP-9 (data not shown). Supernatants obtained from the primary isolates UIVC-IDC-1, UIVC-IDC-4, UIVC-IDC-6, and UIVC-NIDC-1 did not show detectable levels of MMP-7 and MMP-9. Primary culture UIVC-IDC-11 secreted the highest basal level of MMP-7 with 297,006 and 339,296 pg/mL in its correspondent co-culture. UIVC-IDC-11 was also the only primary isolate secreting detectable levels of MMP-9. More interestingly, MMP-1, MMP-2, and MMP-10 showed significant differences between co-cultures and individual 3D cultures (Figure 5). In the case of MMP-1 and -2, the basal levels in individual cultures were average of 19,264 pg/mL (MMP-1) and of 12,474 pg/mL (MMP-2), which in co-culture with monocytes was further increased 3- and 1.9-fold, respectively. For MMP-10, the average level found in supernatants from individual cultures was of 219.3 pg/mL and in the co-cultures with monocytes increased 2.6-fold.

### Primary Breast Cancer Cultures Exhibit an Inflammatory Secretion Profile Rich in Chemotactic Cytokines

**Figure 6:**
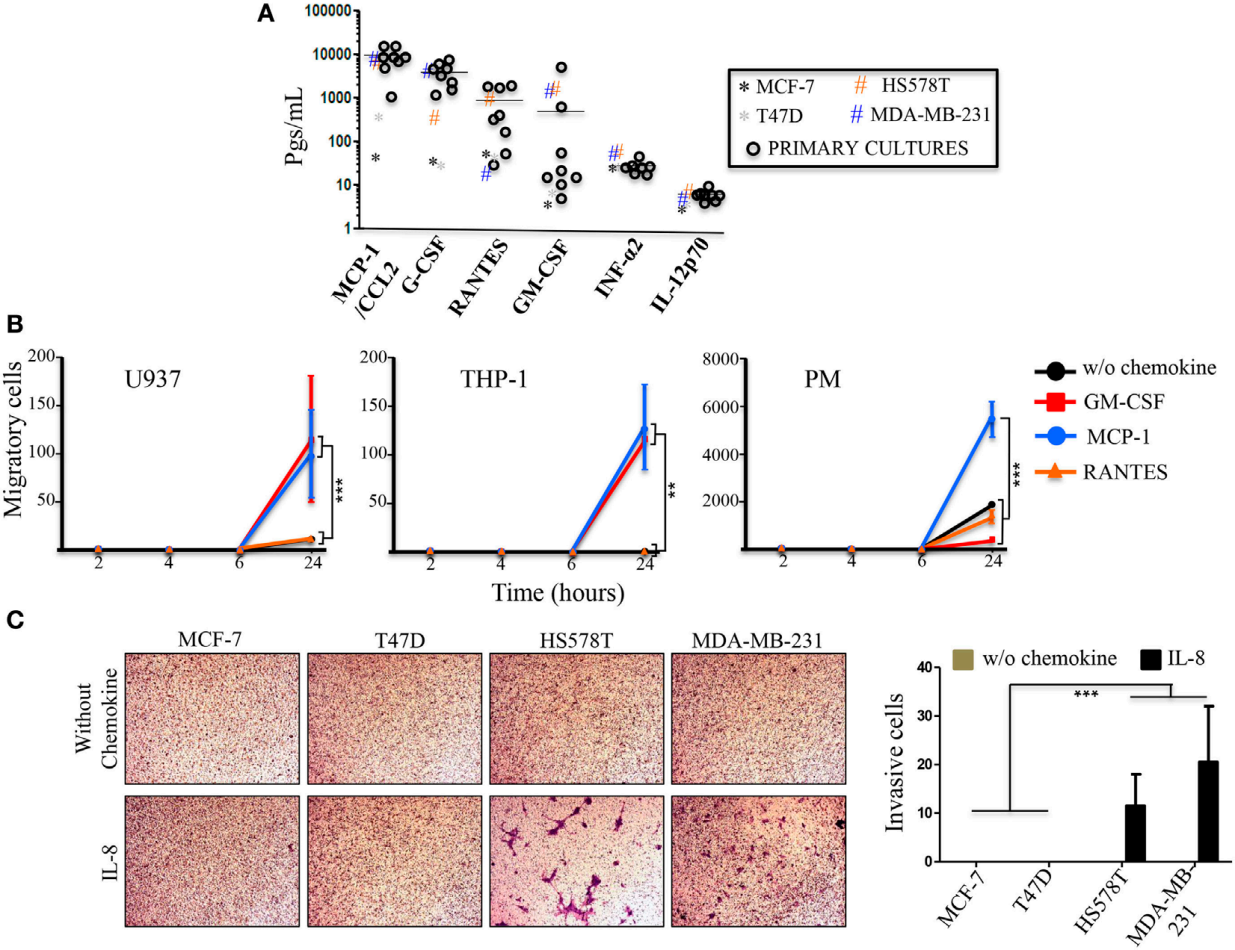
**Primary breast cancer (BrC) cells basal levels of secretion of inflammatory cytokines**. **(A)** The levels of the cytokines, chemokines, and growth factors of interest were determined in the supernatants of the 3D cultures of BrC primary isolates. Each point represents the mean of three determinations made for three independent cultures. The horizontal line represents the average of the primary cultures readings. **(B)** Comparison of the migratory properties of monocytes in response to GM-CSF, MCP-1, and RANTES. **(C)** Invasion assay with the commercial BrC cell lines in response to IL-8. Three independent experiments were performed. Statistical significance is indicated by ***p < 0.001 and **p < 0.01.

In an attempt to understand the cytokine profile of tumor cells that promote an immunological shift or immunoediting in monocytes (24), we analyzed the basal expression levels of the commercial and primary BrC cells (Figure 6A). We found an interesting inflammatory profile promoted by the PC, which consisted of high concentrations of MCP-1/CCL2 (average of 9,277.09 pg/mL), G-CSF/SDF3 (average of 3,999.37 pg/mL) and RANTES (average of 953.19 pg/mL) and, lower levels of INF-α2 (average of 27.07 pg/mL) and IL-12p70 (average of 6.415 pg/mL). For GM-CSF, two cultures were polarized from the rest, UIVC-IDC-1 had 5,022.8 pg/mL and UIVC-IDC-9 had 573.6 pg/mL, the rest of the primary isolates ranged from 4.5 to 51.2 pg/mL. Interestingly, this profile is in good correlation with the profile found in the commercial BrC cell lines, with aggressive cells exhibiting higher levels of MCP-1 and G-CSF, and GM-CSF appreciably separating aggressive from non-aggressive cells. On the contrary, primary and commercial BrC cells showed low levels of INF-α2 and IL-12p70 (Figure 6A). Other cytokines were either or both, not significantly different between aggressive and non-aggressive cells or they had no detectable levels, such as IL-2 and IL-4 (data not shown).

An important observation taken from this experiment was that the most enriched cytokines are critical monocyte chemoattractants. Because of the increased secretion of MMPs, migration assays were performed with commercial and PM in response to GM-CSF, MCP-1, and RANTES in Matrigel filled Transwell inserts. We observed that U937 and THP-1 cells were capable to migrate in response to GM-CSF (average numbers of migrating cells/field were 115 for U937 and 11.5 for THP-1) and to MCP-1 (average numbers of migrating cells/field were 99.7 for U937 and 12.8 for THP-1) and low to null response to RANTES. PM showed high basal migratory properties, giving potent responses to MCP-1 (average number of migrating cells/field of 54,666.7). Although, we observed migration in response to GM-CSF and RANTES the number of migrating cells was not different than the control without chemoattractant (Figure 6B). U937 cells also exhibited migratory properties in the absence of chemoattractant (average number of migrating cells/field 10.7), although lesser than PM (18,666.7 cells). On the contrary, THP-1 cells were not intrinsically migratory in our experimental conditions even without Matrigel.

Interleukin-8 is another potent chemokine. Since IL-8 and MMPs are enriched upon co-culturing of the monocytes and the BrC cell lines, we decided to test whether IL-8 could facilitate invasion of the tumor cells. Figure 6C shows that the aggressive BrC cell lines responded to IL-8 (average number of invading cells/field were 11.5 for HS578T and 20.5 for MDA-MB-231 cells). We did not observe invasive properties in the absence of IL-8 or with the non-aggressive cell lines (Figure 6C). These data argue that the pro-inflammatory profile of the most aggressive BrC cells serve them to attract monocytes, and the BrC-monocyte association further enhances the tumor cell invasive properties.

## Discussion

**Figure 7:**
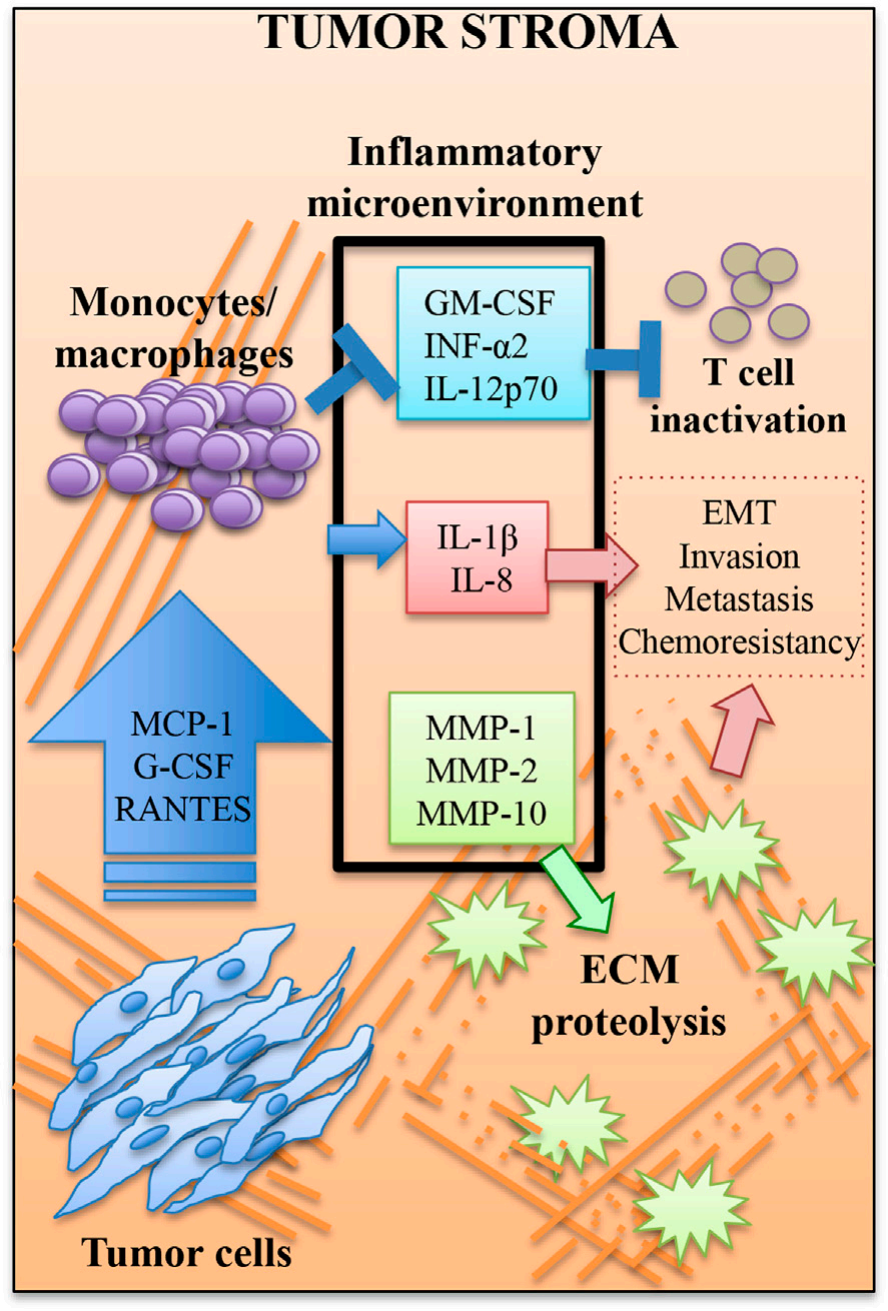
**Working model**. Tumor cells coerce monocytes/macrophages to fulfill activities more attune with tumor grow. MCP-1, G-SCF, and RANTES may be critical components of the message send by tumor cells. Monocytes/macrophages respond helping to establish an inflammatory microenvironment rich in cytokines with pro-tumor activities such as interleukin (IL)-1β and IL-8, which paradoxically may be immunosuppressive of cytotoxic T cell function, since it is high in the previously mentioned cytokines plus GM-CSF and low in INF-α2 and IL-12p70. This inflammatory microenvironment is also enriched of metalloproteinases (MMPs) that promote the degradation of ECM. Altogether, this microenvironment may facilitate the epithelial to mesenchymal transition (EMT), which facilitates invasion and probably metastasis and chemoresistance. The most aggressive tumor cells seem to be more efficient to establish this pro-tumoral microenvironment that further boosts tumor aggressiveness, thus creating a positive feedback loop.

Accurate prognosis and treatment of BrC remains a challenge, partly because most existing classifications of BrC are based on the tumor cell itself, and do not consider all the cell-to-cell interactions within the tumor stroma. In particular, TAMs are considered to be critical for tumor progression. A high density of TAMs indicates a poor prognosis (25–27). To better understand how the tumor cell communicates with recruited monocytes, we have co-cultivated early passages of BrC primary cell isolates and monocytes in a 3D system that recreates the cellular interactions with the ECM. We observed constitutively elevated levels of cytokines MCP-1, G-CSF, and RANTES in tumor cells, and of IL-1β, IL-8, MMP-1, MMP-2, and MMP-10 after co-culture. MCP-1 and G-CSF were potent chemoattractants of monocytes, while IL-8 was a potent inducer of invasion of the aggressive BrC cells (see Figure 7 for a depiction of a working model).

These cytokines are known to contribute toward the establishment of the tumor microenvironment. MCP-1, G-CSF, and RANTES are powerfull chemoattractants of monocytes and of other immune cells, particularly RANTES is a chemoattractant of neutrophils. MCP-1 also acts as an autocrine growth factor for neoplastic cells; promoting angiogenesis, invasion, and metastasis, contributing to tumor growth and dissemination (28, 29). Both MCP-1 and RANTES have minimal expression in non-cancerous epithelial cells and increased expression in neoplastic cells. They contribute toward the establishment of an inflammatory microenvironment, which is paradoxically a potent suppressor of the cytotoxic T cell activity. MCP-1 and RANTES are direct inhibitors of cytotoxic T cells; and G-CSF is in addition associated with the recruitment of myeloid-derived suppressor cells (30, 31). IL-8 and IL-1β are also pro-inflammatory cytokines. IL-1β is produced primarily by activated macrophages and is involved in cell proliferation, differentiation, apoptosis, angiogenesis, and tumor invasion (32–35). IL-1β and EGF act synergistically and induce cell migration and invasion through increased expression and activity of MMPs (36).

Interleukin-8 and G-CSF are powerful stimulators of neutrophil generation, mobilization, activation, and survival (37–39). IL-8 induces multiple intracellular signaling pathways known to be constitutively active in tumor cells, neutrophils, and TAMs. IL-8 is also associated with angiogenesis, proliferation, tumor cell migration, and tumor progression to aggressive stages of the disease (37, 40–44). Fridlender and colleagues have postulated that neutrophils may have N1/N2 polarization states that mirror the M1/M2 phenotypes reported for macrophages; in which, the N2 and M2 types have pro-tumor activities (45). In a mouse model of BrC, Swierczak and colleagues found that blocking the CSF-1 (M-CSF)/CSF-1R interaction to prevent recruitment of circulating monocytes and M2 polarization increased the serum levels of G-CSF, leading to increased neutrophils in the primary tumor, and increased lung metastasis. When a G-CSF receptor antagonistic antibody was administered, it reversed the infiltration of neutrophils and reduced lung metastasis (46). In future studies, it will be interesting to further explore the possible collaboration between tumor cells, monocytes/macrophages and neutrophils; and to assess the capacity of these interactions to suppress T cell function.

In our model, cytokines that inhibit tumorigenesis were found to be suppressed. IL-12p70 and IFN-α2 were downregulated in all PC and commercial BrC cells. GM-CSF was downregulated in six PC, and in MCF-7 and T47D non-aggressive cells. These cytokines are known to have strong anti-tumor activity and are being used as immunoadjuvants in anti-cancer vaccines. Recombinant human INF-α2b is part of an US Food and Drug Administration (FDA)-approved anti-tumor therapy (47–49). IL-12p70 promotes the expansion and activation of cytotoxic CD8 T cells (50, 51) and has been used to stimulate immune anti-tumor activity in animal models (52). IL-12p70 has also been administered in clinical trials to increase the anti-tumor activity of anti-cancer vaccines (53–56). GM-CSF, a potent chemoattractant of neutrophils, monocytes, and lymphocytes (57), has also been used as adjuvant therapy in cancer vaccines. Interestingly, it was found that high GM-CSF doses favored pro-tumor activities, and low doses were an effective immune adjuvant (58).

One of the main mechanisms promoting tumor cell invasion and metastasis is the EMT (59). EMT-promoted cell invasion requires ECM degradation to facilitate cell migration. Mounting evidence support that both IL-1β and IL-8 promote EMT in tumor cells (28, 35, 60–63) and induce tumor cells, and other stromal cells, to secrete MMPs (61, 64–69). MMPs play a key role in the malignant progression of cancer. Their activity degrades and remodels the ECM, and liberates trapped cytokines and growth factor precursors (70–73). In this study, we observed that the levels of MMP-1, MMP-2, and MMP-10 significantly increased in co-cultures of tumor cells/monocytes, and we have previously shown that this correlates with increased collagen degradation (14). High levels of expression of MMP-1 have been associated with tumor growth, invasion and metastasis (74, 75), and MMP-1 levels have been proposed as a marker of poor prognosis in colorectal, breast, and lung cancers (74, 76, 77). MMP-1 has been associated with metastasis to brain in a BrC xenograft model (75). High levels of MMP-2 also correlate with tumor aggressiveness in various types of cancers (78–80). MMP-10 also catalyzes the conversion of pro-MMP-1 into its active form. MMP-10 expression has been associated with invasion and metastasis of pancreatic, cervical, bladder, colorectal, gastric, lung, and breast cancers (81–85).

In summary, cancer aggressiveness is strongly influenced by how tumor cells communicate with other cells within the tumor stroma. Considering that the intra- and inter-tumoral genetic heterogeneity have hampered the design of efficient therapeutic strategies, it is possible that the mechanisms that the tumor uses to communicate with its stroma are more limited and may provide better prognostic and therapeutic targets. Of interest is that the profile of cytokines enriched in tumor cells co-cultured with monocytes better corresponds to the one aided by M1 macrophages, rather than M2 macrophages. This is in agreement with our recent study in which we observed a mix of M1 and M2 types enriched upon monocytes co-culture with tumor cells (15). Interestingly, based on the morphology and behavior in co-culture with monocytes, the primary tumor cells more closely resemble aggressive cell lines. However, most of the patients were classified in clinical stage II and tumor grade II. In agreement, four PC exhibited EMT and invasive properties (Figure S1 in Supplementary Material) (86). We were not able to follow up the patients from which the primary tumor cells were expanded. In future studies it would be very interesting to analyze the nature of the tumor cells in culture (2D and 3D morphology, growth in mesenchymal medium), and their ability to instruct monocytes/macrophages to establish an inflammatory microenvironment rich in cytokines with pro-tumoral functions, and to compare these data with clinical staging and patient survival.

## Author Contributions

EF-P designed the study; GC-R and NE-S performed the experiments; EF-P, GC-R, NE-S, and AM interpreted the results; AM helped to recruit patients and analyzed and classified their type of tumors. EF-P, GC-R, and NE-S wrote the manuscript, and all the authors read it and comment it.

## Conflict of Interest Statement

The authors declare that the research was conducted in the absence of any commercial or financial relationships that could be construed as a potential conflict of interest.

## Supplementary Material

Figure S1 **Primary breast cancer (BrC) cultures exhibit aggressive characteristics**. **(A)** Representative images of the immunofluorescence analysis of EMT markers E-cadherin and vimentin performed to four primary BrC cultures. **(B)** Representative images of the invasion assays and **(C)** graphical representations of the average integrated optical density (IOD) of the invading cells. Scale bars = 100 μM. Magnification 400× for panel **(A)** and 200× for panel **(B)**. Cultures UIVC-IDC-1, UIVC-IDC-4, UIVC-IDC-5, and UIVC-IDC-11 senesced before we could finish these analyses.

## Abbreviations

BrC, breast cancer; PC, primary cultures; PM, primary monocytes; UIVC-IDC or UIVC-NIDC, Unidad de Investigación en Virología y Cáncer- Invasive or Non-invasive ductal carcinoma; MMPs, matrix metalloproteinases; ECM, ectracellular matrix; EMT, epithelial-to-mesenchymal transition; TAMs, tumor-associated macrophages; MDSCs, myeloid-derived suppressor cells; MUC-1, mucin 1 (or epithelial membrane antigen); EpCAM, epithelial cell adhesion molecule.

## References

[1] Ferlay J Soerjomataram I Ervik M Dikshit R Eser S Mathers C GLOBOCAN 2012 v1.0, Cancer Incidence and Mortality Worldwide: IARC CancerBase No. 11 [Internet]. Lyon: International Agency for Research on Cancer (2013). Available from: http://globocan.iarc.fr

[2] Donepudi MS Kondapalli K Amos SJ Venkanteshan P Breast cancer statistics and markers. J Cancer Res Ther (2014) 10(3):506–11. 10.4103/0973-1482.137927 25313729

[3] Ben-Baruch A. Host microenvironment in breast cancer development: inflammatory cells, cytokines and chemokines in breast cancer progression: reciprocal tumor-microenvironment interactions. Breast Cancer Res (2003) 5(1):31–6. 10.1186/bcr554 12559043 PMC154133

[4] Hanahan D Weinberg RA Hallmarks of cancer: the next generation. Cell (2011) 144(5):646–74. 10.1016/j.cell.2011.02.013 21376230

[5] Pollard JW Tumour-educated macrophages promote tumour progression and metastasis. Nat Rev Cancer (2004) 4(1):71–8. 10.1038/nrc1256 14708027

[6] Solinas G Germano G Mantovani A Allavena P. Tumor-associated macrophages (TAM) as major players of the cancer-related inflammation. J Leukoc Biol (2009) 86(5):1065–73. 10.1189/jlb.0609385 19741157

[7] Grivennikov SI Greten FR Karin M Immunity, inflammation, and cancer. Cell (2010) 140(6):883–99. 10.1016/j.cell.2010.01.025 20303878 PMC2866629

[8] Mills CD. Macrophage arginine metabolism to ornithine/urea or nitric oxide/citrulline: a life or death issue. Crit Rev Immunol (2001) 21(5):399–425. 10.1615/CritRevImmunol.v21.i5.10 11942557

[9] Stout RD Jiang C Matta B Tietzel I Watkins SK Suttles J. Macrophages sequentially change their functional phenotype in response to changes in microenvironmental influences. J Immunol (2005) 175(1):342–9. 10.4049/jimmunol.175.1.342 15972667

[10] Gordon S Pluddemann A Martinez Estrada F. Macrophage heterogeneity in tissues: phenotypic diversity and functions. Immunol Rev (2014) 262(1):36–55. 10.1111/imr.12223 25319326 PMC4231239

[11] Lin EY Nguyen AV Russell RG Pollard JW. Colony-stimulating factor 1 promotes progression of mammary tumors to malignancy. J Exp Med (2001) 193(6):727–40. 10.1084/jem.193.6.727 11257139 PMC2193412

[12] Condeelis J Pollard JW. Macrophages: obligate partners for tumor cell migration, invasion, and metastasis. Cell (2006) 124(2):263–6. 10.1016/j.cell.2006.01.007 16439202

[13] Qian BZ Pollard JW. Macrophage diversity enhances tumor progression and metastasis. Cell (2010) 141(1):39–51. 10.1016/j.cell.2010.03.014 20371344 PMC4994190

[14] Chimal-Ramirez GK Espinoza-Sanchez NA Utrera-Barillas D Benitez-Bribiesca L Velazquez JR Arriaga-Pizano LA MMP1, MMP9, and COX2 expressions in promonocytes are induced by breast cancer cells and correlate with collagen degradation, transformation-like morphological changes in MCF-10A acini, and tumor aggressiveness. Biomed Res Int (2013) 2013:279505 10.1155/2013/279505 23762835 PMC3665169

[15] Chimal-Ramirez GK Espinoza-Sanchez NA Chavez-Sanchez L Arriaga-Pizano L Fuentes-Panana EM. Monocyte differentiation towards protumor activity does not correlate with M1 or M2 phenotypes. J Immunol Res (2016) 2016:6031486. 10.1155/2016/6031486 27376091 PMC4916292

[16] Price JE Polyzos A Zhang RD Daniels LM. Tumorigenicity and metastasis of human breast carcinoma cell lines in nude mice. Cancer Res (1990) 50(3):717–21. 2297709

[17] Lacroix M Leclercq G. Relevance of breast cancer cell lines as models for breast tumours: an update. Breast Cancer Res Treat (2004) 83(3):249–89. 10.1023/B:BREA.0000014042.54925.cc 14758095

[18] Prat A Karginova O Parker JS Fan C He X Bixby L Characterization of cell lines derived from breast cancers and normal mammary tissues for the study of the intrinsic molecular subtypes. Breast Cancer Res Treat (2013) 142(2):237–55. 10.1007/s10549-013-2743-3 24162158 PMC3832776

[19] Ziegler E Hansen MT Haase M Emons G Grundker C. Generation of MCF-7 cells with aggressive metastatic potential in vitro and in vivo. Breast Cancer Res Treat (2014) 148(2):269–77. 10.1007/s10549-014-3159-4 25292421

[20] Hass R Bartels H Topley N Hadam M Kohler L Goppelt-Strube M TPA-induced differentiation and adhesion of U937 cells: changes in ultrastructure, cytoskeletal organization and expression of cell surface antigens. Eur J Cell Biol (1989) 48(2):282–93. 2744002

[21] Auwerx J. The human leukemia cell line, THP-1: a multifacetted model for the study of monocyte-macrophage differentiation. Experientia (1991) 47(1):22–31. 10.1007/BF02041244 1999239

[22] Daigneault M Preston JA Marriott HM Whyte MK Dockrell DH. The identification of markers of macrophage differentiation in PMA-stimulated THP-1 cells and monocyte-derived macrophages. PLoS One (2010) 5(1):e8668. 10.1371/journal.pone.0008668 20084270 PMC2800192

[23] Minafra L Di Cara G Albanese NN Cancemi P. Proteomic differentiation pattern in the U937 cell line. Leuk Res (2011) 35(2):226–36. 10.1016/j.leukres.2010.07.040 20801507

[24] Chimal-Ramirez GK Espinoza-Sanchez NA Fuentes-Panana EM Protumor activities of the immune response: insights in the mechanisms of immunological shift, oncotraining, and oncopromotion. J Oncol (2013) 2013:835956 10.1155/2013/835956 23577028 PMC3612474

[25] Medrek C Ponten F Jirstrom K Leandersson K The presence of tumor associated macrophages in tumor stroma as a prognostic marker for breast cancer patients. BMC Cancer (2012) 12:306 10.1186/1471-2407-12-306 22824040 PMC3414782

[26] Lan C Huang X Lin S Huang H Cai Q Wan T Expression of M2-polarized macrophages is associated with poor prognosis for advanced epithelial ovarian cancer. Technol Cancer Res Treat (2013) 12(3):259–67. 10.7785/tcrt.2012.500312 23289476

[27] Sugimura K Miyata H Tanaka K Takahashi T Kurokawa Y Yamasaki M High infiltration of tumor-associated macrophages is associated with a poor response to chemotherapy and poor prognosis of patients undergoing neoadjuvant chemotherapy for esophageal cancer. J Surg Oncol (2015) 111(6):752–9. 10.1002/jso.23881 25752960

[28] Soria G Ofri-Shahak M Haas I Yaal-Hahoshen N Leider-Trejo L Leibovich-Rivkin T Inflammatory mediators in breast cancer: coordinated expression of TNFalpha & IL-1beta with CCL2 & CCL5 and effects on epithelial-to-mesenchymal transition. BMC Cancer (2011) 11:130 10.1186/1471-2407-11-130 21486440 PMC3095565

[29] Li M Knight DA Snyder LA Smyth MJ Stewart TJ. A role for CCL2 in both tumor progression and immunosurveillance. Oncoimmunology (2013) 2(7):e25474. 10.4161/onci.25474 24073384 PMC3782157

[30] Waight JD Hu Q Miller A Liu S Abrams SI. Tumor-derived G-CSF facilitates neoplastic growth through a granulocytic myeloid-derived suppressor cell-dependent mechanism. PLoS One (2011) 6(11):e27690. 10.1371/journal.pone.0027690 22110722 PMC3218014

[31] Agarwal S Lakoma A Chen Z Hicks J Metelitsa LS Kim ES G-CSF promotes neuroblastoma tumorigenicity and metastasis via STAT3-dependent cancer stem cell activation. Cancer Res (2015) 75(12):2566–79. 10.1158/0008-5472.CAN-14-2946 25908586 PMC4470771

[32] Miller LJ Kurtzman SH Anderson K Wang Y Stankus M Renna M Interleukin-1 family expression in human breast cancer: interleukin-1 receptor antagonist. Cancer Invest (2000) 18(4):293–302. 10.3109/07357900009012171 10808364

[33] Lewis AM Varghese S Xu H Alexander HR. Interleukin-1 and cancer progression: the emerging role of interleukin-1 receptor antagonist as a novel therapeutic agent in cancer treatment. J Transl Med (2006) 4:48. 10.1186/1479-5876-4-48 17096856 PMC1660548

[34] Reed JR Leon RP Hall MK Schwertfeger KL. Interleukin-1beta and fibroblast growth factor receptor 1 cooperate to induce cyclooxygenase-2 during early mammary tumourigenesis. Breast Cancer Res (2009) 11(2):R21. 10.1186/bcr2246 19393083 PMC2688950

[35] Li Y Wang L Pappan L Galliher-Beckley A Shi J IL-1beta promotes stemness and invasiveness of colon cancer cells through Zeb1 activation. Mol Cancer (2012) 11:87 10.1186/1476-4598-11-87 23174018 PMC3532073

[36] Ma L Lan F Zheng Z Xie F Wang L Liu W Epidermal growth factor (EGF) and interleukin (IL)-1beta synergistically promote ERK1/2-mediated invasive breast ductal cancer cell migration and invasion. Mol Cancer (2012) 11:79 10.1186/1476-4598-11-79 23083134 PMC3537707

[37] Waugh DJ Wilson C The interleukin-8 pathway in cancer. Clin Cancer Res (2008) 14(21):6735–41. 10.1158/1078-0432.CCR-07-4843 18980965

[38] Geering B Stoeckle C Conus S Simon HU. Living and dying for inflammation: neutrophils, eosinophils, basophils. Trends Immunol (2013) 34(8):398–409. 10.1016/j.it.2013.04.002 23665135

[39] Bendall LJ Bradstock KF. G-CSF: from granulopoietic stimulant to bone marrow stem cell mobilizing agent. Cytokine Growth Factor Rev (2014) 25(4):355–67. 10.1016/j.cytogfr.2014.07.011 25131807

[40] Yuan A Chen JJ Yao PL Yang PC The role of interleukin-8 in cancer cells and microenvironment interaction. Front Biosci (2005) 10:853–65. 10.2741/1579 15569594

[41] Kowanetz M Wu X Lee J Tan M Hagenbeek T Qu X Granulocyte-colony stimulating factor promotes lung metastasis through mobilization of Ly6G+Ly6C+ granulocytes. Proc Natl Acad Sci U S A (2010) 107(50):21248–55. 10.1073/pnas.1015855107 21081700 PMC3003076

[42] Yan HH Pickup M Pang Y Gorska AE Li Z Chytil A Gr-1+CD11b+ myeloid cells tip the balance of immune protection to tumor promotion in the premetastatic lung. Cancer Res (2010) 70(15):6139–49. 10.1158/0008-5472.CAN-10-0706 20631080 PMC4675145

[43] Todorovic-Rakovic N Milovanovic J. Interleukin-8 in breast cancer progression. J Interferon Cytokine Res (2013) 33(10):563–70. 10.1089/jir.2013.0023 23697558 PMC3793647

[44] Chen L Fan J Chen H Meng Z Chen Z Wang P The IL-8/CXCR1 axis is associated with cancer stem cell-like properties and correlates with clinical prognosis in human pancreatic cancer cases. Sci Rep (2014) 4:5911. 10.1038/srep05911 25081383 PMC4118151

[45] Fridlender ZG Sun J Kim S Kapoor V Cheng G Ling L Polarization of tumor-associated neutrophil phenotype by TGF-beta: “N1” versus “N2” TAN. Cancer Cell (2009) 16(3):183–94. 10.1016/j.ccr.2009.06.017 19732719 PMC2754404

[46] Swierczak A Cook AD Lenzo JC Restall CM Doherty JP Anderson RL The promotion of breast cancer metastasis caused by inhibition of CSF-1R/CSF-1 signaling is blocked by targeting the G-CSF receptor. Cancer Immunol Res (2014) 2(8):765–76. 10.1158/2326-6066.CIR-13-0190 25005824

[47] Pichert G Jost LM Fierz W Stahel RA. Clinical and immune modulatory effects of alternative weekly interleukin-2 and interferon alfa-2a in patients with advanced renal cell carcinoma and melanoma. Br J Cancer (1991) 63(2):287–92. 10.1038/bjc.1991.67 1997108 PMC1971775

[48] Jonasch E Haluska FG. Interferon in oncological practice: review of interferon biology, clinical applications, and toxicities. Oncologist (2001) 6(1):34–55. 10.1634/theoncologist.6-1-34 11161227

[49] Asmana Ningrum R. Human interferon alpha-2b: a therapeutic protein for cancer treatment. Scientifica (Cairo) (2014) 2014:970315. 10.1155/2014/970315 24741445 PMC3967813

[50] Schmidt CS Mescher MF. Adjuvant effect of IL-12: conversion of peptide antigen administration from tolerizing to immunizing for CD8+ T cells in vivo. J Immunol (1999) 163(5):2561–7. 10452994

[51] Xiao Z Casey KA Jameson SC Curtsinger JM Mescher MF. Programming for CD8 T cell memory development requires IL-12 or type I IFN. J Immunol (2009) 182(5):2786–94. 10.4049/jimmunol.0803484 19234173 PMC2648124

[52] Lasek W Zagożdżon R Jakobisiak M. Interleukin 12: still a promising candidate for tumor immunotherapy? Cancer Immunol Immunother (2014) 63(5):419–35. 10.1007/s00262-014-1523-1 24514955 PMC3994286

[53] Peterson AC Harlin H Gajewski TF. Immunization with Melan-A peptide-pulsed peripheral blood mononuclear cells plus recombinant human interleukin-12 induces clinical activity and T-cell responses in advanced melanoma. J Clin Oncol (2003) 21(12):2342–8. 10.1200/JCO.2003.12.144 12805336

[54] Trinchieri G. Interleukin-12 and the regulation of innate resistance and adaptive immunity. Nat Rev Immunol (2003) 3(2):133–46. 10.1038/nri1001 12563297

[55] Hamid O Solomon JC Scotland R Garcia M Sian S Ye W Alum with interleukin-12 augments immunity to a melanoma peptide vaccine: correlation with time to relapse in patients with resected high-risk disease. Clin Cancer Res (2007) 13(1):215–22. 10.1158/1078-0432.CCR-06-1450 17200357

[56] Carreno BM Becker-Hapak M Huang A Chan M Alyasiry A Lie WR IL-12p70-producing patient DC vaccine elicits Tc1-polarized immunity. J Clin Invest (2013) 123(8):3383–94. 10.1172/JCI68395 23867552 PMC3726168

[57] Shi Y Liu CH Roberts AI Das J Xu G Ren G Granulocyte-macrophage colony-stimulating factor (GM-CSF) and T-cell responses: what we do and don’t know. Cell Res (2006) 16(2):126–33. 10.1038/sj.cr.7310017 16474424

[58] Parmiani G Castelli C Pilla L Santinami M Colombo MP Rivoltini L. Opposite immune functions of GM-CSF administered as vaccine adjuvant in cancer patients. Ann Oncol (2007) 18(2):226–32. 10.1093/annonc/mdl158 17116643

[59] Foroni C Broggini M Generali D Damia G. Epithelial-mesenchymal transition and breast cancer: role, molecular mechanisms and clinical impact. Cancer Treat Rev (2012) 38(6):689–97. 10.1016/j.ctrv.2011.11.001 22118888

[60] Li XJ Peng LX Shao JY Lu WH Zhang JX Chen S As an independent unfavorable prognostic factor, IL-8 promotes metastasis of nasopharyngeal carcinoma through induction of epithelial-mesenchymal transition and activation of AKT signaling. Carcinogenesis (2012) 33(7):1302–9. 10.1093/carcin/bgs181 22610073 PMC3405654

[61] Leibovich-Rivkin T Liubomirski Y Bernstein B Meshel T Ben-Baruch A. Inflammatory factors of the tumor microenvironment induce plasticity in nontransformed breast epithelial cells: EMT, invasion, and collapse of normally organized breast textures. Neoplasia (2013) 15(12):1330–46. 10.1593/neo.131688 24403855 PMC3884524

[62] Singh JK Simoes BM Clarke RB Bundred NJ. Targeting IL-8 signalling to inhibit breast cancer stem cell activity. Expert Opin Ther Targets (2013) 17(11):1235–41. 10.1517/14728222.2013.835398 24032691

[63] Wang L Tang C Cao H Li K Pang X Zhong L Activation of IL-8 via PI3K/Akt-dependent pathway is involved in leptin-mediated epithelial-mesenchymal transition in human breast cancer cells. Cancer Biol Ther (2015) 16(8):1220–30. 10.1080/15384047.2015.1056409 26121010 PMC4622725

[64] Barille S Akhoundi C Collette M Mellerin MP Rapp MJ Harousseau JL Metalloproteinases in multiple myeloma: production of matrix metalloproteinase-9 (MMP-9), activation of proMMP-2, and induction of MMP-1 by myeloma cells. Blood (1997) 90(4):1649–55. 9269785

[65] Inoue K Slaton JW Eve BY Kim SJ Perrotte P Balbay MD Interleukin 8 expression regulates tumorigenicity and metastases in androgen-independent prostate cancer. Clin Cancer Res (2000) 6(5):2104–19. 10815938

[66] Voronov E Shouval DS Krelin Y Cagnano E Benharroch D Iwakura Y IL-1 is required for tumor invasiveness and angiogenesis. Proc Natl Acad Sci U S A (2003) 100(5):2645–50. 10.1073/pnas.0437939100 12598651 PMC151394

[67] Li A Varney ML Valasek J Godfrey M Dave BJ Singh RK. Autocrine role of interleukin-8 in induction of endothelial cell proliferation, survival, migration and MMP-2 production and angiogenesis. Angiogenesis (2005) 8(1):63–71. 10.1007/s10456-005-5208-4 16132619

[68] Han J Bae SY Oh SJ Lee J Lee JH Lee HC Zerumbone suppresses IL-1beta-induced cell migration and invasion by inhibiting IL-8 and MMP-3 expression in human triple-negative breast cancer cells. Phytother Res (2014) 28(11):1654–60. 10.1002/ptr.5178 24890258

[69] Yang CM Hsieh HL Yu PH Lin CC Liu SW IL-1beta induces MMP-9-dependent brain astrocytic migration via transactivation of PDGF receptor/NADPH oxidase 2-derived reactive oxygen species signals. Mol Neurobiol (2015) 52(1):303–17. 10.1007/s12035-014-8838-y 25159478

[70] Hotary KB Allen ED Brooks PC Datta NS Long MW Weiss SJ. Membrane type I matrix metalloproteinase usurps tumor growth control imposed by the three-dimensional extracellular matrix. Cell (2003) 114(1):33–45. 10.1016/S0092-8674(03)00513-0 12859896

[71] Pardo A Selman M. MMP-1: the elder of the family. Int J Biochem Cell Biol (2005) 37(2):283–8. 10.1016/j.biocel.2004.06.017 15474975

[72] Iida J McCarthy JB. Expression of collagenase-1 (MMP-1) promotes melanoma growth through the generation of active transforming growth factor-beta. Melanoma Res (2007) 17(4):205–13. 10.1097/CMR.0b013e3282a660ad 17625450

[73] Lu X Wang Q Hu G Van Poznak C Fleisher M Reiss M ADAMTS1 and MMP1 proteolytically engage EGF-like ligands in an osteolytic signaling cascade for bone metastasis. Genes Dev (2009) 23(16):1882–94. 10.1101/gad.1824809 19608765 PMC2725946

[74] Sauter W Rosenberger A Beckmann L Kropp S Mittelstrass K Timofeeva M Matrix metalloproteinase 1 (MMP1) is associated with early-onset lung cancer. Cancer Epidemiol Biomarkers Prev (2008) 17(5):1127–35. 10.1158/1055-9965.EPI-07-2840 18483334

[75] Liu H Kato Y Erzinger SA Kiriakova GM Qian Y Palmieri D The role of MMP-1 in breast cancer growth and metastasis to the brain in a xenograft model. BMC Cancer (2012) 12:583. 10.1186/1471-2407-12-583 23217186 PMC3526403

[76] Murray GI Duncan ME O’Neil P Melvin WT Fothergill JE. Matrix metalloproteinase-1 is associated with poor prognosis in colorectal cancer. Nat Med (1996) 2(4):461–2. 10.1038/nm0496-461 8597958

[77] Poola I DeWitty RL Marshalleck JJ Bhatnagar R Abraham J Leffall LD. Identification of MMP-1 as a putative breast cancer predictive marker by global gene expression analysis. Nat Med (2005) 11(5):481–3. 10.1038/nm1243 15864312

[78] Schmalfeldt B Prechtel D Harting K Spathe K Rutke S Konik E Increased expression of matrix metalloproteinases (MMP)-2, MMP-9, and the urokinase-type plasminogen activator is associated with progression from benign to advanced ovarian cancer. Clin Cancer Res (2001) 7(8):2396–404. 11489818

[79] Brehmer B Biesterfeld S Jakse G. Expression of matrix metalloproteinases (MMP-2 and -9) and their inhibitors (TIMP-1 and -2) in prostate cancer tissue. Prostate Cancer Prostatic Dis (2003) 6(3):217–22. 10.1038/sj.pcan.4500657 12970724

[80] Pellikainen JM Ropponen KM Kataja VV Kellokoski JK Eskelinen MJ Kosma VM. Expression of matrix metalloproteinase (MMP)-2 and MMP-9 in breast cancer with a special reference to activator protein-2, HER2, and prognosis. Clin Cancer Res (2004) 10(22):7621–8. 10.1158/1078-0432.CCR-04-1061 15569994

[81] Gill JH Kirwan IG Seargent JM Martin SW Tijani S Anikin VA MMP-10 is overexpressed, proteolytically active, and a potential target for therapeutic intervention in human lung carcinomas. Neoplasia (2004) 6(6):777–85. 10.1593/neo.04283 15720804 PMC1550316

[82] Goodison S Chang M Dai Y Urquidi V Rosser CJ. A multi-analyte assay for the non-invasive detection of bladder cancer. PLoS One (2012) 7(10):e47469. 10.1371/journal.pone.0047469 23094052 PMC3477150

[83] Urquidi V Goodison S Cai Y Sun Y Rosser CJ. A candidate molecular biomarker panel for the detection of bladder cancer. Cancer Epidemiol Biomarkers Prev (2012) 21(12):2149–58. 10.1158/1055-9965.EPI-12-0428 23097579 PMC3537330

[84] Rosser CJ Ross S Chang M Dai Y Mengual L Zhang G Multiplex protein signature for the detection of bladder cancer in voided urine samples. J Urol (2013) 190(6):2257–62. 10.1016/j.juro.2013.06.011 23764080 PMC4013793

[85] Zhang G Miyake M Lawton A Goodison S Rosser CJ. Matrix metalloproteinase-10 promotes tumor progression through regulation of angiogenic and apoptotic pathways in cervical tumors. BMC Cancer (2014) 14:310. 10.1186/1471-2407-14-310 24885595 PMC4022983

[86] Lango-Chavarria M Chimal-Ramirez GK Ruiz-Tachiquin ME Espinoza-Sanchez NA Suarez-Arriaga MC Fuentes-Panana EM. A 22q11.2 amplification in the region encoding microRNA-650 correlates with the epithelial to mesenchymal transition in breast cancer primary cultures of Mexican patients. Int J Oncol (2017) 50(2):432–40. 10.3892/ijo.2017.3842 28101578 PMC5238778

