# Corrigendum: IL-1β, IL-8, and Matrix Metalloproteinases-1, -2, and -10 Are Enriched upon Monocyte–Breast Cancer Cell Cocultivation in a Matrigel-Based Three-Dimensional System

**DOI:** 10.3389/fimmu.2017.00856

**Published:** 2017-07-27

**Authors:** Nancy Adriana Espinoza-Sánchez, Gloria Karina Chimal-Ramírez, Alejandra Mantilla, Ezequiel Moisés Fuentes-Pananá

**Affiliations:** ^1^Unidad de Investigación en Virología y Cáncer, Hospital Infantil de México Federico Gómez, Ciudad de México, México; ^2^Programa de Doctorado en Ciencias Biomédicas, Facultad de Medicina, Universidad Nacional Autónoma de México, Ciudad de México, México; ^3^Departamento de Patología, Hospital de Oncología, Centro Médico Nacional Siglo XXI, Instituto Mexicano del Seguro Social, Ciudad de México, México

**Keywords:** breast cancer, monocytes, matrigel-based 3D culture, inflammation, IL-1β, IL-8, MMPs

Corrigendum on: **Figure 6 | Primary breast cancer (Brc) cells basal levels of secretion of inflammatory cytokines**.

**Figure 6:**
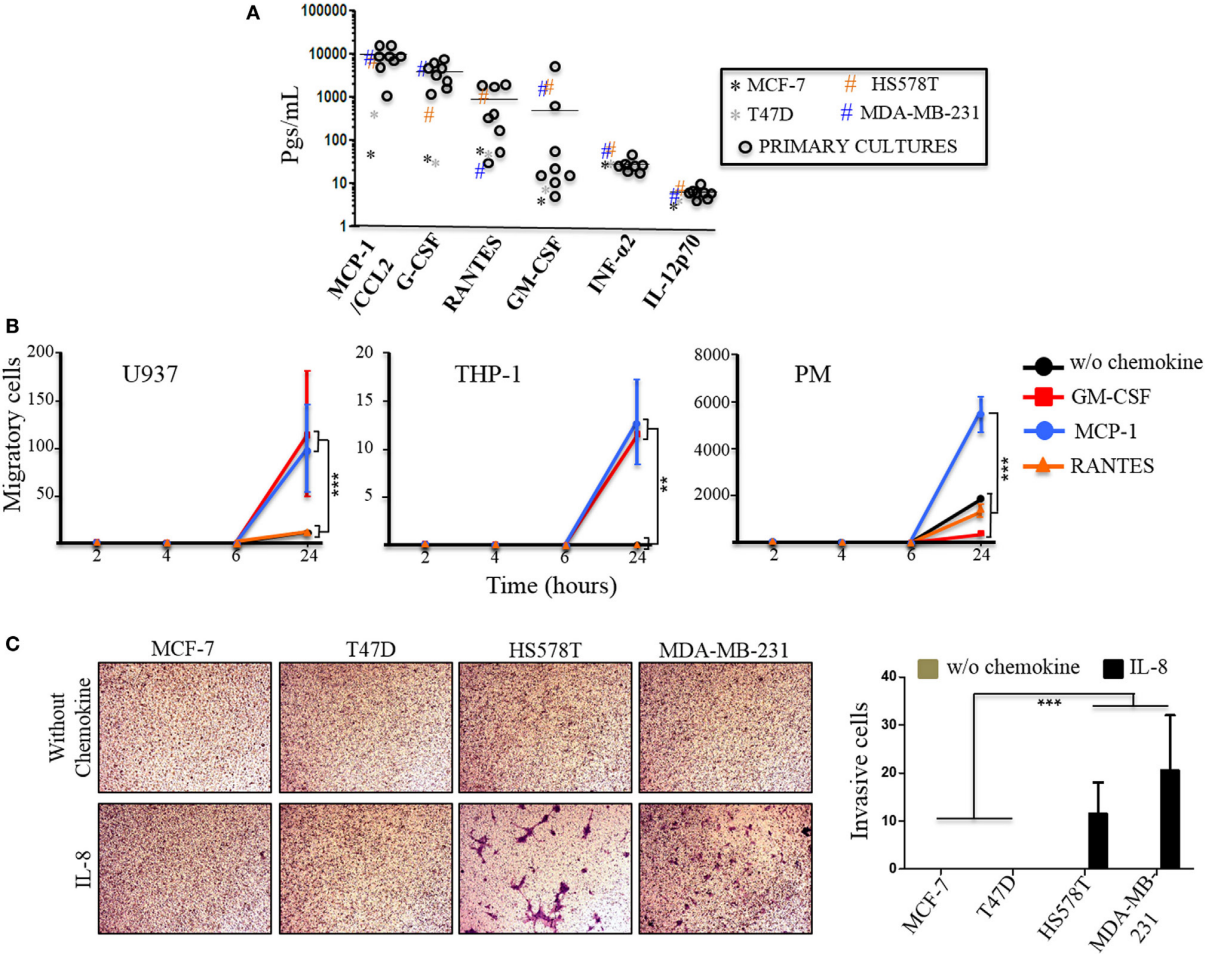
Primary breast cancer (BrC) cells basal levels of secretion of inflammatory cytokines. **(A)** The levels of the cytokines, chemokines, and growth factors of interest were determined in the supernatants of the 3D cultures of BrC primary isolates. Each point represents the mean of three determinations made for three independent cultures. The horizontal line represents the average of the primary cultures readings. **(B)** Comparison of the migratory properties of monocytes in response to GM-CSF, MCP-1, and RANTES. **(C)** Invasion assay with the commercial BrC cell lines in response to IL-8. Three independent experiments were performed. Statistical significance is indicated by ***p < 0.001 and **p < 0.01.

In the original article, there was a mistake in the Figure 6, **panel B** as published. **The scale in the Y axes is incorrect**. The corrected appears below: should be 20, 15, 10 and 5, instead of 200, 150, 100 and 50. The authors apologize for this error and state that this does not change the scientific conclusions of the article in any way.

## Conflict of Interest Statement

The authors declare that the research was conducted in the absence of any commercial or financial relationships that could be construed as a potential conflict of interest.

