# Supplementary figures and images for: IL-1β, IL-8, and Matrix Metalloproteinases-1, -2, and -10 Are Enriched upon Monocyte–Breast Cancer Cell Cocultivation in a Matrigel-Based Three-Dimensional System
Source: Front Immunol. 2017 Mar 8;8:205. doi: 10.3389/fimmu.2017.00205 (PMC5340783; doi:10.3389/fimmu.2017.00205)

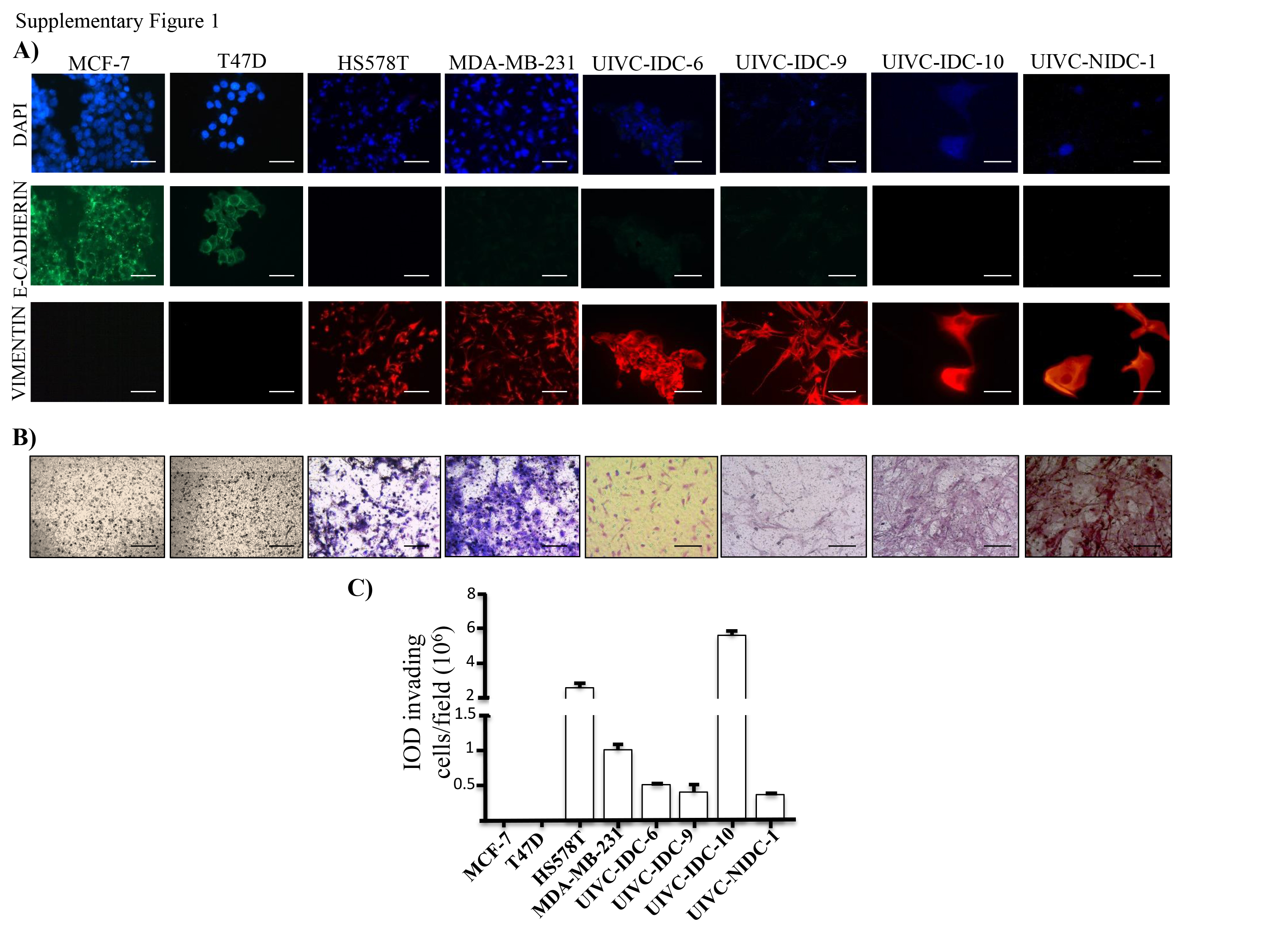

Supplement: Figure S1 — Primary breast cancer (BrC) cultures exhibit aggressive characteristics. (A) Representative images of the immunofluorescence analysis of EMT markers E-cadherin and vimentin performed to four primary BrC cultures. (B) Representative images of the invasion assays and (C) graphical representations of the average integrated optical density (IOD) of the invading cells. Scale bars = 100 μM. Magnification 400× for panel (A) and 200× for panel (B). Cultures UIVC-IDC-1, UIVC-IDC-4, UIVC-IDC-5, and UIVC-IDC-11 senesced before we could finish these analyses. [file Image_1.JPG]
